# New perspectives on patient expectations of treatment outcomes: results from qualitative interviews with patients seeking complementary and alternative medicine treatments for chronic low back pain

**DOI:** 10.1186/1472-6882-14-276

**Published:** 2014-07-30

**Authors:** Clarissa Hsu, Karen J Sherman, Emery R Eaves, Judith A Turner, Daniel C Cherkin, DeAnn Cromp, Lisa Schafer, Cheryl Ritenbaugh

**Affiliations:** Group Health Research Institute, Seattle, USA; University of Arizona, Tucson, AZ USA; Department of Psychiatry & Behavioral Sciences, University of Washington School of Medicine, Seattle, WA USA; Department of Rehabilitation Medicine, University of Washington School of Medicine, Seattle, WA USA

## Abstract

**Background:**

Positive patient expectations are often believed to be associated with greater benefits from complementary and alternative medicine (CAM) treatments. However, clinical studies of CAM treatments for chronic pain have not consistently supported this assumption, possibly because of differences in definitions and measures of expectations. The goal of this qualitative paper is to provide new perspectives on the outcome expectations of patients prior to receiving CAM therapies for chronic low back pain.

**Methods:**

We conducted semi-structured interviews with 64 individuals receiving massage, chiropractic, acupuncture or yoga for chronic low back pain. Interviews were recorded and transcribed. Transcripts were analyzed by a team of experienced qualitative researchers using an immersion/crystallization approach to coding and analysis.

**Results:**

Overall, participants’ expectations of treatment outcomes tended to cluster in four key domains: pain relief, improved function (including an increase in ability to engage in meaningful activities), improved physical fitness, and improved overall well-being (including mental well-being). Typically, patients had modest expectations for outcomes from treatment. Furthermore, outcome expectations were complex on several levels. First, the concept of expectations overlapped with several related concepts; in particular, hopes. Participants sometimes used expectations and hopes interchangeably and at other times made clear distinctions between these two terms depending on context. A related finding was that participants were cautious about stating that they expected positive outcomes. Finally, participants articulated strong interrelationships among the four key domains and often discussed how changes in one domain might affect other domains.

**Conclusions:**

Overall, these findings contribute to a growing body of literature exploring the role of expectations in patient outcomes. This paper provides important guidance that may help refine the way treatment expectations are studied in the future. In particular, participants’ statements indicate that standardized measures of patient expectations should include items that capture hesitancy to articulate overly optimistic outcomes as well as interrelationships among different outcomes.

## Background

Patient expectations are believed to play an important role in the “placebo effect” [1, 2]. Positive outcomes of complementary and alternative medicine (CAM) therapies are often attributed largely, if not entirely, to nonspecific effects associated with patients’ high expectations of benefits [3–5]. This attribution persists even though many people seeking these therapies do not have prior experience or cultural knowledge of them [6]. Surprisingly, little research has focused on the expectations patients have when beginning CAM therapies. Studies by Bishop and colleagues have touched on expectations in the context of broader research questions about reasons to start or continue CAM therapies, but do not focus on expectations and exploring them in depth [7–9]. Clinical trial findings have been inconsistent regarding associations of patient expectations with their treatment outcomes [10–15]. At least some of these inconsistencies could be due to a lack of standard methodology for assessing patient expectations [11]. As a starting point for improving consistency in measuring expectations, in-depth qualitative analysis of the ways patients articulate and conceptualize their expectations is needed. A qualitative approach might provide critical insights into what patients expect when beginning new treatments and what, therefore, is useful to measure.

Patient expectations are complex and likely influenced by a number of factors [16] such as sociodemographic characteristics [17]; prior experience (e.g., classical conditioning) [1, 2]; support or skepticism on the part of friends and family [18, 19]; and therapeutic interactions [2, 20]. Further, hopes may be a central factor in determining how patients assess and report their expectations, although this factor has been inadequately considered in research on patient expectations [21–23].

The goal of this study is to collect and examine qualitative data from patients to increase our understanding of their expectations and hopes about novel CAM therapies for chronic low back pain. We conducted semi-structured qualitative interviews focused on patient expectations, first with CAM providers [24] and then with patients seeking one of four CAM therapies (acupuncture, yoga, chiropractic, massage). The qualitative study was one phase in a project to develop a questionnaire to measure treatment expectations among patients with chronic low back pain. This paper focuses on patient expectations about treatment outcomes before or early in treatment. Change in patients’ expectations over the course of treatment will be the topic of an in-depth analysis in a separate forthcoming manuscript.

Initial coding of interview data contributed to the design of questionnaire items for further testing in cognitive interviews [25]; the resulting draft questionnaire is currently undergoing psychometric analysis. Here, we report on our in-depth analysis of qualitative interviews with patients to better understand how patients conceptualize and articulate expectations and hopes for the outcomes of CAM treatments for which they have limited or no prior experience.

## Methods

### Study sample and procedures

Interviews were conducted with 64 participants (23 in Tucson, AZ and 41 in Seattle, WA) from January through September 2011. We enrolled patients with chronic low back pain (defined as having less than two weeks without pain in the last three months) who were receiving one of four CAM treatments for chronic low back pain: chiropractic, acupuncture, massage or yoga. These four CAM treatments were chosen because they are the most common non-pharmacological CAM therapies used for back pain [26]. There is moderate-to-good evidence for their effectiveness in clinical trials [27]. Inclusion of multiple therapies increased the likelihood that the findings might be applicable to other CAM and non-pharmacological therapies. We enrolled 24 participants prior to their first treatment session, 12 shortly after their first treatment session, eight after two or more treatment sessions (but within several weeks of starting treatment), and 20 after they had been in treatment for several months or longer. In Tucson, we made additional efforts to recruit patients from Hispanic and other ethnic/racial minority groups to capture a diversity of perspectives.

Participants were recruited using a convenience sample of individuals who found out about the study through CAM provider offices, a research website, or an online advertisement. Recruitment materials specified our interest in people with chronic low back pain that were planning to try a CAM therapy that they had not previously used for chronic low back pain. Individuals interested in participating contacted the study team via telephone and were screened for eligibility criteria: 1) adult (aged 20 to 70 years), 2) reported chronic low back pain as evidenced by persistent low back pain present in both the past week and for at least three months, 3) rated the average bothersomeness of their low back pain as 3 or more on a 0–10 scale, 4) rated pain interference with daily activities as 3 or more on a 0–10 scale, 5) not pregnant, 6) did not have symptoms consistent with sciatica, and 7) committed to trying or recently started a CAM therapy not used previously for chronic low back pain or had used a CAM therapy for chronic low back pain in the last 5 years. All interested people who met the eligibility criteria were invited to participate. For those eligible, telephone or in-person interviews were conducted as soon as feasible by one of four experienced interviewers (CH, ERE, LS, AH). Both interviewers in Tucson were fluent in Spanish.

For study participants enrolled prior to or within 2 weeks of beginning the CAM treatment, three interviews were conducted: 1) before the initial treatment session or as soon as possible after the initial session; 2) two to three weeks after the initial session; and 3) three months after the initial session. Participants who had been using the treatment for several weeks before enrolling were interviewed twice: an initial interview and a follow-up at three months. Participants who had been using the CAM treatment for more than two months were interviewed only once. Table 1 shows the number of participants recruited in each timeframe by type of treatment and geographic location.

**Table 1:** **Participants by recruitment site and timeframe relative to beginning treatment**

	Yoga		Chiropractic		Massage		Acupuncture		Total	
	Seattle	Tucson	Seattle	Tucson	Seattle	Tucson	Seattle	Tucson	Seattle	Tucson
1^st^ interview prior to treatment (3 interviews)	9	1	3	0	5	1	3	2	20	4
1^st^ interview within 1 week after 1^st^ treatment (3 interviews)	2	0	1	3	2	0	3	1	8	4
1^st^ interview several weeks after start of treatment, (2 interviews)	1	0	1	0	2	0	4	0	8	0
Interviewed after an extended period using the treatment (1 interview)	0	5	4	3	1	2	0	5	5	15
Total	12	6	9	6	10	3	10	8	41	23

Interviewers used a semi-structured interview guide to ensure that similar questions and themes were addressed in all interviews. However, interviewers were free to adapt the questions, probe responses, and follow respondent-driven topics. Questions were developed before conducting interviews using a conceptual model of patient expectations based on an extensive review of the literature (described in an earlier publication) [24]. Study participants also provided information on their age, gender, race, and ethnicity. All but 12 interviews were conducted by phone. Five interviews were conducted in Spanish. Interviews lasted 20–60 minutes, with follow-up interviews typically shorter than first interviews. All interviews were audio-recorded and transcribed for analysis. Patients were compensated $90 for their participation (dispersed over the number of interviews completed) in Seattle and $30 for the first interview plus $20 for each follow-up interview in Tucson. All study materials, consent forms and protocols were reviewed and approved by the institutional review boards at Group Health Research Institute in Seattle and the University of Arizona in Tucson. All study participants provided informed consent and were assured that their names would be kept confidential. All names in this manuscript are pseudonyms assigned by the research team.

### Data analysis

Interview transcripts were analyzed thematically by three of the interviewers (CH, ERE, LS) and another analysis team member (DC) using the immersion/crystallization approach, which emphasizes gaining an in-depth knowledge of the data to identify key themes [28]. Data collection and analysis were conducted sequentially. The analysis team drafted a coding scheme based on the conceptual model [24], discussion of findings, and initial impressions from the data. Other team members reviewed the draft coding scheme and suggested revisions. All four members of the analysis team independently coded two transcripts, then compared and discussed the coding to reconcile differences and add new codes for themes that emerged from the data. Revisions to the coding scheme were made as needed. This process was repeated three times, at which point the analysis team concluded that the coding scheme included most of the themes that were relevant to the study (both aprior and emergent themes) and agreement about code definitions was sufficient for high consistency across coders. One team member in Seattle (DC) coded the remaining transcripts for interviews conducted in Seattle and one interviewer in Tucson (ERE) coded the remaining transcripts for the interviews conducted in Tucson. Although the coding scheme was revised as needed over the course of coding, only minor changes were made after the three rounds of coding by all four coders. We used qualitative analysis software (Atlas.ti) to code transcripts and facilitate analyses [29].

One key element of the coding scheme was a set of codes designating whether a statement represented a patient view prior to treatment or after the initiation of CAM treatment (“pre” for prior and “post” for during or after treatment). These codes were assigned based on both the timing of the interview and context of the question and patient response. For participants interviewed after treatment start, responses to interview questions that asked them to reflect on their expectations prior to treatment were coded “pre”. This approach allowed the examination of expectations prior to treatment, even though in some cases these expectations were recalled after the fact.

Interviews resulted in a complex data set with information on a wide array of patients’ expectations, experiences, characteristics and attitudes. Our analyses for this paper focused on a subset of codes pertaining to patients’ pre-treatment expectations about key treatment outcomes. For the purposes of this analysis we used a broad definition of expectations, including references to desired or hoped-for outcomes. The broad perspective allowed full capture and understanding of the contexts and variations in how participants conceptualized their expectations. Coded data were reviewed multiple times by the primary author (CH). Prevalent subthemes were summarized in a coding memo—a document with brief descriptions of key findings with numerous quotes that illustrate the findings. To guide additional analysis and reach consensus on findings, the entire team discussed several iterations of the coding memo with.

## Results

### Participant characteristics

Interviews were conducted with 64 individuals (48 women, 16 men) with chronic low back pain. Table 2 summarizes key participant characteristics.

**Table 2:** **Participant characteristics**

	Seattle	Arizona	Total
**Type of treatment**			
Chiropractic	9	6	15
Acupuncture	10	8	18
Massage	10	3	13
Yoga	12	6	18
	41	23	64
**Gender**			
Female	30	18	48
Male	11	5	16
**Race/Ethnicity**			
Non-Hispanic White	31	11	42
Hispanic	2	6	8
Asian	4	1	5
Black/African American	1	1	2
Alaska Native/American Indian	1	0	1
Unknown	2	3	5

### Overarching issues related to treatment outcome expectations

Our analysis primarily focused on how patients articulated their expectations regarding CAM treatment outcomes prior to experiencing the treatment (for those who had begun treatment, this was based on recall). We found that the words “expectation” and “expect” had numerous meanings depending on the context in which they were used. Participants often used these terms inconsistently. It was not uncommon for the same participant in the same interview to use the terms differently in different contexts. Participants often used a variety of words or phrases when talking about expectations, including expect, realistically expect, think, believe, and hope. These terms may show how a number of concepts such as hope, belief, and being realistic influenced participants’ explanations of their expectations. Furthermore, we found that expectations often changed over time as participants gained more experience with the treatment and their chronic low back pain problems evolved.

### Hope versus expectations

Almost all participants queried about their expectations spontaneously responded with comments, both about what they expected and what they hoped for.

I’m hoping that long term that this will lessen my pain and give me a better quality of life. That’s what I’m hoping for. But I’m not going in with an expectation that this is what’s going to happen. (Leslie, chiropractic patient, Seattle)

Although some participants seemed to use “hope” and “expect” as synonyms, when asked directly most made clear distinctions between the two concepts. These distinctions were remarkably consistent across participants. Hope was described as the most optimistic/fanciful end of the outcome range and expectations as the most realistic projections of what might happen based on prior experience and illness history.

Oh, I think realistically, I don’t think it’ll change much. I would hope that it would help, I hope I would have some reduction in the amount of pain that I have, especially at this moment. (Kris, yoga student, Seattle)

### No expectations

A small number of participants reported having no expectations for their treatment (although many of those still reported hopes). Some of these participants were concerned that having expectations could lead to disappointment.

I guess my hope would be that the uncomfort in my back is gone . . . but honestly I try not to have any expectations. Because if it doesn’t work then that’s not very much fun, to have a bunch of expectations and it doesn’t work. (Sarah, acupuncture patient, Seattle)

Reluctance to express expectations is another example of the complex relationship between hopes and expectations, since several participants who stated that they had “no expectations” were willing to articulate their hopes.

Well, I hoped it would work or help, for my sake as well as my husband’s, but as far as what I expected, that was totally different, I didn’t expect anything, I expected nothing, nothing one way or another. (Catherine, acupuncture patient, Tucson)

Reluctance to express expectations may indicate the power that participants placed in the act of articulating or giving voice to their expectations. Invoking expectations was viewed as inherently risky. Articulated expectations introduce the possibility of being disappointed, and might invoke a more archaic fear of “jinxing needs oneself by boasting or being too positive”. Similar to the tradition of “knocking on wood” (i.e., “this is going well, knock on wood”), participants may have been reluctant to speak about a positive future because of concern about jeopardizing future positive outcomes.

I learned long ago that expectations usually get me into trouble, so I try not to use expectations. I was in a 12-Step program, such as Alcoholics Anonymous, I have two sons who are into drugs. And expectations of what they should be and how they should treat me and that sort of thing, just led me into trouble. I would say things that I really had no business saying, I would try to get into their business when it wasn’t my business and so, you know, I had [Crying] excuse me, sorry. I had expectations of a long life with my husband and he died, so…I tried to be just realistic. (Jean, acupuncture patient, Tucson)

The few participants who said that they had neither hopes nor expectations attributed this to inexperience with or lack of confidence in the treatment.

Yeah, I mean I guess there was nothing that I really expected, just try to keep an open mind about it, but I was definitely a little like, “OK, what’s this gonna be like?” But really just wanting to learn something new and wanting to make sure it was in an environment that supported, kind of my level and my . . . injuries or issues . . . I know that there’s all kinds of poses and instruction and things like that, but other than that I really didn’t know what to expect. (Roger, yoga student, Seattle)

Well, right, I said I had no expectation really. And I guess I didn’t have any hope really. I mean I hoped my back would get better, but not necessarily -- I didn’t believe that acupuncture would help it. (Daniel, acupuncture patient, Seattle)

### Complete symptom resolution/“cure”

Expectations of complete resolution or cure were rare. When we examined all mentions of “cure” or text related to complete recovery, we found very few instances in which study participants articulated expectations of complete pain relief as a result of the CAM treatment they were seeking. The few patients who did mention the possibility of complete symptom relief generally framed this thought in terms of hopes rather than expectations that their pain might be eliminated.

My hope would be for my back pain to be relieved or maybe eradicated that would be great. My expectation is that it could be improved, but not necessarily eradicated. (Adam, acupuncture patient, Seattle)

Many participants expressed the belief that their back pain was a long-term problem.

Maybe it, you know, comes with age or whatever having done things for a number of years, I have no pie in the sky expectation that, oh, we’ll suddenly find something, totally gone, not a problem. I just want to be able to manage it and decrease the amount of time that it hurts, the duration of the hurt. (Jillian, massage patient, Seattle)

A few patients who were already using CAM treatments before entering our study reported that they originally expected that the treatment would make the pain go away or “fix” them. The participant below expressed the hope that she would find an alternative therapy that would provide permanent pain relief. She was careful to point out, however, that she was aware that others, primarily her doctors, believed that she was not being “realistic.”

Um, I think that they don’t think I’m really uh, realistic. Especially with this. . . . I mean I respect and love doctors and I’m very grateful for them but I just feel like they don’t know it all. Especially the more I just look at different situations as far as holistic health. I think that there’s something out there for me that’ll work. I don’t just want to accept the fact that I’m in pain. I don’t- and I don’t want to cover it up with drugs. I want it to be fixed. Something is wrong if I’m hurting and I want it fixed. (Kayla, acupuncture patient, Tucson)

### Key domains of outcome expectations

In addition to examining the ways in which participants articulated their expectations of CAM treatments, we examined how they talked about expectations regarding treatment outcomes. Based on the results of our analysis, we grouped outcome expectations into four general domains—pain, function, physical fitness, and mood/quality of life/sleep/wellness. With the exception of the physical fitness domain, expectations in these domains were similar across the different CAM treatments.

### Expectations-pain

Pain relief was the outcome expectation most frequently discussed (either spontaneously or when probed) by the participants we interviewed.

Well I would hope-the bottom line is that I want to be relieved of the pain that I have. I would say I don’t have expectations beyond the current pain . . . (Harry, acupuncture patient, Seattle)

However, expectations regarding pain relief were often modest. Participants generally did not expect their pain to go away, but instead talked about “relief” or pain “subsiding,” “decreasing,” or “lessening.”

I didn’t think it would eliminate my pain, I just thought it would help the healing and help, you know, me be more comfortable, but it wouldn’t make it go away. (Greg, massage patient, Seattle)

### Expectations-function and participation in meaningful activities

Many participants talked about expectations for functional outcomes, generally articulated as the ability to do meaningful activities, which often focused around work, hobbies, social life, or activities of daily living.

Oh, I would do a lot more walking and a lot more physical things and more yard work, more being with my dogs. I have Basset Hounds so they’re all short. You mostly need to get on the floor with them. And, you know, and I can get down on the floor, it’s getting back up that just brings tears to my eyes and I want to be able to do that. (Nora, chiropractic patient, Seattle)

I’m hoping that just little things, like I can do the walk around the little water pond with my grandkids so they can feel like I’m a part of what they’re doing, you know? I mean I don’t want to go run a marathon, I don’t think I’ll do that anytime soon, I never ran before I got sick, you know what I mean? Just the little things, day to day, being able to vacuum, and clean the bathrooms on the same day, I can’t do that right now, I just want to be able to do, to complete that task, ‘cause I’ve been to the point five years ago where my daughter had to move in with me to dress me because I couldn’t dress myself. (Angie, chiropractic patient, Tucson)

### Expectations-physical fitness

Some participants talked about expectations for improvements in their physical fitness, focusing most frequently on muscle strength, flexibility, and overall fitness. We found that, with few exceptions, expectations for increased flexibility, strength and other aspects of physical fitness were mentioned by participants using yoga. Although the link between expectations of physical fitness outcomes with yoga seems common sense, it nevertheless points to an important domain of outcome expectations.

Interviewer: And what effects do you think the yoga will have on your pain?

Participant: Well, I’m hoping that I’ll gain more strength in my core, so that I can support myself better to make myself stronger, to hopefully prevent me from, you know, tweaking my back, I guess. So, I’m just hoping that it will provide me with a little more strength to support my back so that I can do things like vacuum the house or just whatever without just I guess decreasing the risk of triggering the back pain from coming back as often as it has been lately. (Roger, yoga student, Seattle)

Interviewer: To take things back before you went to the yoga for the first time, how did you think your life might change as a result of doing the yoga?

Participant: Well, I was thinkin’ that it’ll, like make me more flexible and make me more healthy because I’ll be more flexible and I’ll get better toned and it’ll just be an all around benefit to me for my health. (Jackie, yoga student, Seattle)

Interviewer: So when you started the treatments, did you expect your life to change very much?

Participant: Yeah, in the sense of become healthier, lose weight, um, be able to exercise more . . . (Phil, acupuncture patient, Tucson)

### Expectations-mood/quality of life/well-being

Another emergent domain of outcomes focused around mood, energy and overall quality of life. Expectations under this domain included positive changes in: mood, stress, energy, overall quality of life, sleep, and well-being.

I think it would just help the overall not—or trying not—to slide into being depressed about it. Not have to use up so much strength and energy just to marshal all my horses to carry on even though I hurt so much. (Jillian, massage patient, Seattle)

You know, I think that my life would improve because I am so irritable, it’s just kind of bad. It makes me sad that, yeah, it’s really depressing sometimes, I mean I normally wouldn’t be, and so I think I would just be in a more peaceful place. (Kim, yoga student, Seattle)

I was hoping that it could basically allow me to restore my daily routine and quality of life as it was before the acute episode happened. (Joan, chiropractic patient, Seattle)

### Interrelationships among domains

Although we explore the four outcome domains separately, many of the quotes demonstrate the ways in which patients see these domains as interrelated. The connection between pain and function was one of the most commonly mentioned relationships between domains. The following quote, which is typical of many in the dataset, illustrates how participants linked improvements in pain with increased function.

Well I’m hoping it will provide a more permanent relief than what I’m getting now. My expectation is that the pain will subside if not go away entirely. I don’t know how long it’s going to take but I would hope that I can become more active, can return to the active lifestyle that I’ve had. I’ve climbed mountains and have been pretty active and I can’t do that now because I just can’t even go on a hike it’s too painful. (Harry, acupuncture patient, Seattle)

Pain was not the only domain that affected other domains. Increased flexibility was often associated with decreased pain. Stress was characterized as a cause of pain, therefore its decrease could lead to reduced pain and improved function. Relationships were also noted between pain and sleep. Participants noted that pain led to difficulty sleeping, and conversely, that trouble sleeping was a contributor to increased pain.

Although changes in pain were often cited as leading to many other outcomes, participants also saw that other domains might affect pain. In the following example, a participant described how better sleep might lead to decreased pain.

I’m just hoping that I can find a way to if not manage the pain, maybe she can help me figure out ways to relax so that I can sleep better because I’m really exhausted and I think that it’s sort of a cycle where the not sleeping makes the pain worse and then when the pain is worse, you can’t sleep. So I know that they deal a lot with relaxation techniques and I’m hoping that I can at least take that away if the pain doesn’t go away. (Heidi, massage patient, Seattle)

Another patient connected increased energy with increased participation in activities, regardless of pain reduction:

Well, I imagine that I’ll have more energy, more lust for life. Have more desire to do more things. Things that I’m not able to do now. Things that I enjoy. Ride a bike. [chuckles] (Paula, massage patient, Tucson)

The participant below reported an expectation that yoga will improve her mobility in general, which could lead to increased motivation for physical activity and subsequently, decreased back pain.

I think that [yoga] would just help more with my mobility and it wouldn’t be such a nuisance if I were to maybe start practicing yoga on more of a, maybe doing it a few times a week. As opposed to just not doing any kind of activity, rather than just going to work and doing what I do and not stretching and not exercising for that matter, I feel that it probably could help a lot, just with my mobility in that general area and probably like, you know, make me, just more comfortable with moving and not having that continual, “Oh my back!” You know? It feels so stiff all the time. (Patricia, yoga student, Seattle)

These examples illustrate various ways that participants articulated the interrelationship between the outcome domains, demonstrating the multifaceted and dynamic nature of the outcome expectations. Participants viewed their back pain as part of a complex mind-body system in which changes in one domain could have multiple impacts on other domains.

## Discussion

We found that participants seeking one of four CAM therapies reported complex and varied expectations when seeking CAM. We grouped participants’ expectations regarding treatment outcomes in several key domains: pain; function (including participation in meaningful activities); physical fitness; and mood, quality of life, and well-being. However, participants seeking CAM did not conceptualize treatment outcomes as discrete or isolated domains, but rather as interrelated, which has important implications for how to appropriately characterize or measure their treatment expectations. Although participants were most likely to expect decreases in pain to lead to changes in other outcome domains, this was not universal and some articulated the expectations that improvements in other outcome domains could lead to decreased pain.

Additionally, the way that people talked about their expectations was highly nuanced, especially in the distinction some people made between hopes and expectations. Our findings are consistent with those of Haanstra et al., who found that patients talked about both “values (what they hoped) and probabilities (what they thought likely)” [30].

Overall, expectations for pain reduction were modest. Participants rarely expressed expectations for “cure” or complete symptom resolution as primary goals of seeking treatment, although a few maintained some hope for this. Those who did say they *hoped* for pain elimination often followed such statements with expressions of more modest expectations and admitted that often, their hopes were perceived as not “realistic” by significant others or health care providers. A few participants who reported having “no expectations” mentioned that having expectations would merely set them up for disappointment. Remaining “open” to the possibility of cure, while carefully mitigating against the possibility of despair is a commonly expressed position for individuals living with chronic pain or illness [18, 21, 23].

In our study, many responses acknowledged the power of forming and articulating hopes and expectations, and the pros and cons of anticipating a future improved state. Participants were careful to articulate “realistic” expectations. Others scholars have demonstrated that patients often express concerns about having overly optimistic expectations that could lead to disappointment [21, 23, 31] or even negatively influence (jinx) future outcomes [31]. In general, patients may be ambivalent about voicing hopes or expectations. Simpson, exploring ways that hope can make patients vulnerable, states: “part of what makes hope hope is the awareness that not every hope is realized” [19]. Among our respondents, this vulnerability was even more salient for expectations, which were considered more concrete and therefore carried greater risk of disappointment if not realized. Although beyond the scope of this analysis, our findings suggest that the power of words in making something real may impact the way individuals formulate and verbalize hopes or expectations. Social scientists widely acknowledge that words and discourse have the power to shape our reality [32–35]. Patients’ ambivalence and inconsistency in articulating hopes and expectations are important to consider in future research related to treatment expectations. The extent to which individuals might be reluctant to acknowledge hopes and expectations because of fears of future disappointment, or conversely, might overestimate hopes and expectations with the intent of manifesting a desired reality, is an area that needs additional exploration.

A key implication of these findings is the need for more nuanced ways of characterizing and assessing patient expectations. Standardized questionnaires measuring expectations prior to beginning a CAM treatment for chronic low back pain might more fully capture the range of important dimensions of expectations with questions that ask about both hopes and realistic expectations, including for specific outcomes such as pain, function/participation in meaningful activities, physical fitness, mood, and general well-being. Furthermore, our findings might offer insights about other CAM therapies commonly used to treat chronic pain-related conditions. Our findings may translate to other pain conditions with a vague or unknown biophysical explanation but a broad impact on people’s lives, such as fibromyalgia. However, this study has a more limited application to cancer and other terminal illnesses for which patient expectations have been the topic of considerable research. Expectations and hopes for cancer treatment often focus on forestalling death and/or the possibility of being cured [36] whereas the participants in our study focused on incremental improvements in pain, function, physical health and mood, quality of life, and well-being. Additional work is needed to explore the applicability of these findings on a broader range of symptoms, conditions and/or illness categories.

The limitations of this study include that the findings are based on interviews with a volunteer sample recruited through CAM providers and online advertising in two geographic areas. This might have led to sample selection bias or a sample that might not reflect the full range of experience or variability of a larger, national sample. However, we found that similar themes emerged across the two sites. Another limitation is that some participants received CAM therapies prior to the interview and we depended on retrospective accounts of their expectations. However, we saw similar themes in pre-treatment outcome expectations irrespective of whether participants had already engaged in treatment. Despite these limitations, the results provide valuable insights into the nuanced and complex ways that participants articulate expectations of treatment outcomes when starting a CAM treatment for chronic low back pain.

## Conclusion

Overall, these findings contribute to the growing body of literature exploring the role of expectations on patient outcomes. We found that participant expectations when seeking CAM treatments for chronic low back pain were modest, calling for a reexamination of hypotheses that high expectations are responsible for positive treatment outcomes for CAM modalities. Furthermore, we found outcome expectations were complex on several levels. First, the concept of expectations overlapped with several related concepts, in particular, hopes. Participants sometimes used the terms ‘expectations’ and ‘hopes’ interchangeably and at other times made clear distinctions between these terms depending on context. A related finding was that most participants were hesitant to articulate optimistic expectations for their treatment outcomes. This may reflect a number of issues including lack of knowledge/experience with CAM treatments, past experiences of persistent pain, and concern about the power and risk of acknowledging a desired future state. Finally, we found that although participants’ outcome expectations could be clustered in several defined domains, participants viewed these domains as interrelated Our results indicate that standardized measures of patient treatment expectations should include items that acknowledge patient hesitancy to articulate overly optimistic expectations and recognize patient views about the interrelationships among different outcomes. Our findings provide novel and nuanced insights into patients’ expectations regarding CAM treatments for chronic low back pain. These findings can be used to expand and improve upon research on the nature and impact of patient expectations on the lived experiences and outcomes of CAM treatments.

## Abbreviations

CAM: Complementary and alternative medicine.
